# The burden of urinary incontinence and its association with knowledge, practices, and comorbidities among women in Madinah, Saudi Arabia: a cross-sectional study

**DOI:** 10.3389/fmed.2025.1726741

**Published:** 2026-01-12

**Authors:** Abdulaziz Bakhsh, Hazim Alnazawi, Osama Alsehli, Mohammed Almuzaini, Hussam Aloufi, Ahmed Alahmadi, Ziyad Alharbi, Taher Mohammed, Walaa Abdullah Mumena, Emad Rajih, Waleed H. Mahallawi

**Affiliations:** 1 General and Specialized Surgery Department, College of Medicine, Taibah University, Madinah, Saudi Arabia; 2 Urology Section, Department of Surgery, King Faisal Specialist Hospital and Research Center, Madinah, Saudi Arabia; 3 Urology Department, King Fahad General Hospital, Madinah, Saudi Arabia; 4 Urology Department, Prince Mohammad Bin Abdulaziz Hospital, Madinah, Saudi Arabia; 5 Urology Department, King Salman Bin Abdulaziz Medical City, Madinah, Saudi Arabia; 6 Dermatology Department, King Fahad Hospital, Al-Baha, Saudi Arabia; 7 Clinical Nutrition Department, College of Applied Medical Sciences, Taibah University, Madinah, Saudi Arabia; 8 Clinical Laboratory Sciences Department, College of Applied Medical Sciences, Taibah University, Madinah, Saudi Arabia; 9 Health and Life Research Center, Taibah University, Madinah, Saudi Arabia

**Keywords:** cross-sectional study, health practices, knowledge, prevalence, risk factors, Saudi Arabia, urinary incontinence, women's health

## Abstract

**Background:**

Urinary incontinence (UI) is a prevalent and debilitating condition that significantly impairs quality of life. While risk factors are well-documented globally, data on its prevalence, associated knowledge, and related practices among women in specific regions of Saudi Arabia remain scarce.

**Objective:**

This study aimed to determine the prevalence of UI, assess women's knowledge and practices regarding the condition, and identify associated sociodemographic and clinical factors among women in Madinah, Saudi Arabia.

**Methods:**

A community-based, cross-sectional online survey was conducted between June and August 2024 among 394 Saudi women aged 25–65 years in Madinah, recruited via convenience sampling through social media platforms (WhatsApp, X, and Telegram). A self-administered questionnaire collected data on sociodemographics, health characteristics, UI prevalence (over the past 6 months), and a 16-item knowledge and 4-item practices scale. Statistical analyses included descriptive statistics, Mann–Whitney U/Kruskal–Wallis tests, and binary logistic regression to identify factors associated with UI.

**Results:**

The prevalence of UI was 30% (*n* = 118). Among the total sample, symptoms suggestive of stress UI were most common (52.8%), followed by urge (35.8%), mixed (34.3%), and overflow UI (19.0%). Participants demonstrated moderate general knowledge (mean score: 8.95 ± 2.30 out of 16) but markedly low understanding of specific UI types (e.g., only 10.9% knew the difference between stress and urge UI). Negative health-seeking practices were reported, including neglecting treatment due to embarrassment (16.8%) and hesitancy to consult a doctor (19.3%). Logistic regression revealed that older age (46–55 years: OR = 3.22, 95% CI: 1.57–6.60; 56–65 years: OR = 3.41, 95% CI: 1.23–9.44), being married (OR = 2.61, 95% CI: 1.19–5.73), overweight (OR = 1.96, 95% CI: 1.11–3.47), obesity (OR = 2.97, 95% CI: 1.66–5.32), constipation (OR = 2.20, 95% CI: 1.30–3.72), chronic cough (OR = 3.67, 95% CI: 1.52–8.85), diuretic use (OR = 2.75, 95% CI: 1.09–6.95), hypercholesterolemia (OR = 2.41, 95% CI: 1.45–3.99), history of UTI (OR = 2.34, 95% CI: 1.48–3.92), and more negative practices (OR = 2.94, 95% CI: 2.31–3.72) were significant independent predictors of UI. Knowledge score was not a significant predictor.

**Conclusion:**

UI is a common condition among women in Madinah, strongly associated with a profile of demographic and clinical factors. The critical gap in specific UI knowledge and the prevalence of negative health-seeking behaviors highlight an urgent need for public health campaigns that address these specific deficits and routine, sensitive screening for high-risk groups to bridge the treatment gap.

## 1 Introduction

Urinary incontinence (UI), the complaint of any involuntary leakage of urine, represents a significant global public health concern with profound physical, psychological, and social consequences (1, 2). It is estimated to affect hundreds of millions of women worldwide, with prevalence rates varying widely based on population characteristics, definitions, and cultural contexts (3, 4). The condition is not life-threatening, but it severely compromises quality of life, leading to social isolation, depression, anxiety, and substantial economic burdens on healthcare systems (5, 6, 23).

The etiopathogenesis of UI is multifactorial, involving a complex interplay of anatomical, physiological, neurological, and lifestyle factors (7, 8). Established risk factors include advanced age, obesity, vaginal childbirth, menopause, chronic constipation, chronic respiratory conditions causing cough, and certain surgical procedures (9, 10). Furthermore, comorbidities such as diabetes mellitus and hypertension have been implicated in its pathophysiology (11, 12).

Despite its high prevalence and impact, UI remains under-reported and under-treated, often shrouded in silence and stigma (13, 14). Many women perceive UI as an inevitable part of aging or childbirth and are often unaware of the effective treatment options available, ranging from conservative management (lifestyle interventions, pelvic floor muscle training) to pharmacotherapy and surgery (15, 16). This lack of knowledge directly influences health-seeking behaviors, leading to delays in diagnosis and management (17, 18).

In the Kingdom of Saudi Arabia, evolving demographics and lifestyle changes, including an aging population, rising rates of obesity, and a high prevalence of chronic conditions such as diabetes and hypertension, are contributing to an increased burden of non-communicable diseases (19, 20). Given that these are established risk factors for UI, this shifting health profile may significantly influence its epidemiology and burden in the region. Previous Saudi studies have reported UI prevalence ranging from 30% to over 50% in specific female cohorts, highlighting it as a common issue (21, 22, 40). However, most research has been concentrated in major urban centers like Riyadh and Jeddah. The city of Madinah, a major cultural and religious hub with a unique sociodemographic profile, remains understudied in this context.

Moreover, while some studies have assessed prevalence, comprehensive data on the specific knowledge gaps and subsequent health practices related to UI among Saudi women are limited. Understanding these dimensions is crucial for developing effective, culturally sensitive public health interventions. Therefore, this study aimed to (1) determine the current prevalence and types of UI among women in Madinah, crucial for developing targeted public health strategies in this unique sociodemographic setting, (2) assess their knowledge and practices regarding UI, and (3) identify the sociodemographic and clinical factors independently associated with UI in this population.

## 2 Materials and methods

### 2.1 Study design and population

This community-based, cross-sectional study was conducted between June 1 and August 30, 2024, among post-pubertal Saudi women aged 25–65 years residing in Madinah, Saudi Arabia. Non-Saudi nationals, non-residents of Madinah, men, and individuals who provided incomplete survey responses were excluded. Ethical approval was granted by the Research Ethical Committee at Taibah University, Madinah (IRB No: TU-24-9-112; date: March 1, 2024). Electronic informed consent was obtained from all participants before they proceeded to the survey questionnaire.

### 2.2 Sample size and sampling technique

The sample size was calculated using the OpenEpi tool, version 3. Assuming a UI prevalence of 35% based on previous Saudi studies (21, 22), a 95% confidence level, and a 5% margin of error, a minimum sample of 350 participants was required. We targeted 384 participants to enhance the study's precision and account for potential exclusions. A total of 446 initial responses were collected. A convenience sampling method was employed, disseminating the online survey primarily through popular social media platforms (WhatsApp, X, and Telegram) along with a brief explanation of the study's objectives. This involved sharing the survey link directly in relevant groups and channels. It was not a formal snowball sampling technique where participants were asked to recruit others.

### 2.3 Data collection tool and measures

Data were collected using a structured, self-administered electronic questionnaire developed on the Google Forms platform. The questionnaire was constructed based on an extensive review of the relevant literature (16, 24) and was reviewed for content validity by a panel of three urologists. A pilot study was conducted with 30 women (who were then excluded from the main study) to assess the clarity, comprehensibility, and internal consistency of the tool. Based on the pilot feedback, minor wording changes were made to improve clarity. The knowledge scale demonstrated good reliability with a Cronbach's alpha of >0.7.

The questionnaire comprised four sections:

1. *Sociodemographic and clinical characteristics:*

Including age, marital status, education level, smoking history, weight, height (to calculate BMI), and history of specific medical conditions (menopause, constipation, chronic cough, diabetes, hypertension, hypercholesterolemia, UTI) and procedures (use of diuretics, hip surgery, abdominal surgery).

2. *Urinary incontinence assessment:*

Participants were asked if they had experienced any involuntary urine leakage in the past 6 months (yes/no). Those responding “yes” were further questioned about the presence of symptoms suggestive of stress, urge, overflow, and mixed UI based on standardized definitions (25) (e.g., symptoms of stress UI: leakage with coughing/sneezing; symptoms of urge UI: leakage preceded by a sudden, strong desire to void).

3. *Knowledge scale:*

A 16-item scale assessed knowledge about UI's nature, types, and treatment options. Responses were “True,” “False,” or “I don't know.” Correct answers were scored 1, and incorrect/“don't know” answers were scored 0, yielding a total knowledge score ranging from 0 to 16.

4. *Practices scale:*

A 4-item scale evaluated negative behaviors related to UI, including reduced activity participation, neglect due to embarrassment, hesitation to buy protective products, and hesitation to seek medical help. Each affirmative response was scored 1, creating a total practices score from 0 to 4, with higher scores indicating more negative health-seeking practices.

### 2.4 Statistical analysis

Data analysis was performed using IBM SPSS Statistics for Windows, Version 20 (IBM Corp., Armonk, NY, USA). Descriptive statistics for continuous variables were presented as mean ± standard deviation (SD) and median with interquartile range (IQR). Categorical variables were summarized as frequencies and percentages (%). As the knowledge and practice scores are ordinal data derived from summed Likert-type items, non-parametric tests were deemed appropriate. The Mann–Whitney U and Kruskal–Wallis tests were used to compare these scores across different categorical groups. Binary logistic regression analysis was performed to identify factors independently associated with the presence of UI, calculating odds ratios (OR) with 95% confidence intervals (CI). All analyses were two-tailed, and a *p*-value of < 0.05 was considered statistically significant.

## 3 Results

### 3.1 Sample characteristics

Of the 446 initial responses, 52 were excluded (for being male, non-Saudi, or living outside Madinah), resulting in 394 participants for the final analysis. The mean age of the participants was 44.3 ± 8.72 years, with a median of 45.0 years (IQR: 39.0–50.0). The mean BMI was 28.0 ± 5.68 kg/m^2^, indicating, on average, an overweight population. The majority of women were married (86.8%, *n* = 342), held a university degree (86.2%, *n* = 340), and had never smoked (97.0%, *n* = 382). A significant proportion (68.5%, *n* = 270) were overweight or obese. Other relevant clinical characteristics included menopause (30.7%, *n* = 121), constipation (18.3%, *n* = 72), and a history of UTI (23.1%, *n* = 91). The detailed sample characteristics are presented in Table 1.

**Table 1:** Sociodemographic and clinical characteristics of the study participants (*n* = 394).

**Variable**	***n***	**%**
**Age groups**		
25–35 years	71	18.0
36–45 years	138	35.0
46–55 years	159	40.4
56–65 years	26	6.60
**Marital status**		
Not married	52	13.2
Married	342	86.8
**Education level**		
< High school	8	2.00
High school	46	11.7
Bachelor	315	79.9
Postgraduate	25	6.30
**Smoking history**		
Never smoked	382	97.0
Previous or current smoker	12	3.00
**Weight status**		
Underweight (BMI < 18.5 kg/m^2^)	5	1.30
Healthy weight (BMI = 18.5–24.9 kg/m^2^)	119	30.2
Overweight (BMI = 25.0–29.9 kg/m^2^)	151	38.3
Obesity (BMI ≥ 30.0 kg/m^2^)	119	30.2
**Menopause**		
No	273	69.3
Yes	121	30.7
**Experienced constipation**		
No	322	81.7
Yes	72	18.3
**Experienced chronic cough**		
No	372	94.4
Yes	22	5.60
**Used diuretics**		
No	375	95.2
Yes	19	4.80
**Drink a lot of tea and coffee**		
No	187	47.5
Yes	207	52.5
**Performed hip surgery**		
No	341	86.5
Yes	53	13.5
**Performed a surgical procedure in the abdominal region**		
No	278	70.6
Yes	116	29.4
**Diagnosed with diabetes mellitus**		
No	347	88.1
Yes	47	11.9
**Diagnosed with hypertension**		
No	341	86.5
Yes	53	13.5
**Diagnosed with hypercholesterolemia**		
No	313	79.4
Yes	81	20.6
**History of UTI**		
No	303	76.9
Yes	91	23.1

### 3.2 Prevalence and types of urinary incontinence

Within the six months preceding the survey, 30% (*n* = 118) of the participants reported experiencing UI. Among the total sample of 394 women, symptoms suggestive of the various UI types were reported as follows: stress UI was the most frequently reported (52.8%, *n* = 208), followed by urge UI (35.8%, *n* = 141), mixed UI (34.3%, *n* = 135), and overflow UI (19.0%, *n* = 75). Among the 118 women with UI, the distribution of reported types was stress UI (67.8%, *n* = 80), urge UI (46.6%, *n* = 55), mixed UI (40.7%, *n* = 48), and overflow UI (22.0%, *n* = 26). The percentages exceed 100% as participants could report symptoms indicative of multiple UI types.

### 3.3 Knowledge and practices related to urinary incontinence

The overall knowledge score had a mean of 8.95 ± 2.30 (median: 9.00). As detailed in Table 2, there were notable disparities in knowledge across different items. While a high percentage of women knew that UI can be managed through exercise or nutrition (81.5%), that medical (81.2%) and natural (85.3%) treatments are available, knowledge about the specific types of UI was markedly low. Only 10.9% correctly recognized the difference between stress and urge UI, and a mere 5.3% knew what “mixed UI” entailed.

**Table 2:** Knowledge and practices related to urinary incontinence among participants (*n* = 394).

**Serial**		***n***	**%**
**Knowledge related to urinary incontinence**			
1	UI is a temporary problem, no	55	14.0
2	UI can be treated medically, yes	300	76.1
3	UI is a natural result after childbirth, yes	205	52.0
4	UI is a natural result of aging, yes	293	74.4
5	Normal activities, including light exercises, are affected by UI, yes	154	39.1
6	UI restricts social activities, yes	308	78.2
7	UI can be managed through physical exercise or healthy nutrition, yes	321	81.5
8	There are several types of UI, yes	305	77.4
9	Recognized the difference between stress UI and urge UI, yes	43	10.9
10	Recognized what “mixed UI” is, yes	21	5.30
11	Recognized what “overflow UI” is, yes	40	10.2
12	Methods for treating UI include lifestyle changes, yes	310	78.7
13	Natural treatments are available for UI, yes	336	85.3
14	Medical treatments are available for UI, yes	320	81.2
15	Surgical treatments are an option for UI, yes	257	65.2
16	All options are correct (items 12–15), yes	259	65.7
**Practices toward urinary incontinence**			
1	UI resulted in decreased participation in activities, yes	65	16.5
2	Neglected UI due to embarrassment, yes	66	16.8
3	Hesitated to buy protective products for UI, yes	61	15.5
4	Hesitated to go to the doctor for UI treatment, yes	76	19.3

Regarding information sources, 53.0% (*n* = 209) of women reported receiving information about UI, primarily through social media (41.6%), with only 22.1% receiving information from healthcare professionals.

The practices score had a mean of 0.68 ± 1.22 (median: 0.00). A concerning 19.3% of women hesitated to seek medical treatment for UI, 16.8% neglected the problem due to embarrassment, and 15.5% hesitated to buy protective products.

### 3.4 Association of sample characteristics with knowledge and practices

As shown in Table 3, knowledge scores were not significantly associated with any of the sociodemographic or clinical characteristics (*p* > 0.05 for all). In contrast, practices scores (indicating more negative behaviors) were significantly associated with several factors. These included older age (*p* = 0.002), being married (*p* = 0.030), higher weight status (*p* < 0.001), menopause (*p* = 0.026), experiencing constipation (*p* < 0.001) or chronic cough (*p* = 0.022), having undergone hip surgery (*p* = 0.039), and being diagnosed with diabetes (*p* = 0.026), hypertension (*p* = 0.011), hypercholesterolemia (*p* < 0.001), or a history of UTI (*p* < 0.001).

**Table 3:** Association between participant characteristics and knowledge/practice scores (*n* = 394).

**Variable**	**Knowledge related to UI score out of 16**	**Practices related to UI scores out of 4**
**Age groups**		
25–35 years	8.96 ± 2.17	0.39 ± 1.04
	9.00 (7.00–10.0)	0.00 (0.00–0.00)
36–45 years	9.07 ± 2.24	0.54 ± 1.09
	9.00 (7.75–11.0)	0.00 (0.00–0.25)
46–55 years	8.92 ± 2.17	0.90 ± 1.36
	9.00 (8.00–11.0)	0.00 (0.00–2.00)
56–65 years	8.50 ± 2.18	0.88 ± 1.24
	9.00 (7.00–10.0)	0.00 (0.00–2.00)
*p*-value	0.549	0.002
**Marital status**		
Not married	8.92 ± 2.49	0.33 ± 0.83
	9.00 (7.00–11.0)	0.00 (0.00–0.00)
Married	8.96 ± 2.28	0.73 ± 1.26
	9.00 (7.75–11.0)	0.00 (0.00–1.00)
*p*-value	0.812	0.030
**Education level**		
< High-school	8.75 ± 4.13	0.13 ± 0.35
	10.0 (6.50–12.0)	0.00 (0.00–0.00)
High-school	8.48 ± 2.65	0.93 ± 1.36
	8.00 (7.00–10.25)	0.00 (0.00–2.00)
Bachelor	9.01 ± 2.17	0.69 ± 1.23
	9.00 (8.00–11.0)	0.00 (0.00–1.00)
Postgraduate	9.20 ± 2.60	0.32 ± 0.85
	10.00 (8.00–11.0)	0.00 (0.00–0.00)
*p*-value	0.503	0.116
**Smoking history**		
Never smoked	8.95 ± 2.31	0.68 ± 1.23
	9.00 (7.00–11.0)	0.00 (0.00–1.00)
Previous or current smoker	9.08 ± 1.98	0.67 ± 0.99
	8.00 (8.00–10.75)	0.00 (0.00–2.00)
*p*-value	0.892	0.832
**Weight status**		
Underweight/healthy weight (BMI < 24.9 kg/m^2^)	9.05 ± 2.41	0.32 ± 0.90
	9.00 (7.25–11.0)	0.00 (0.00–0.00)
Overweight (BMI = 25.0–29.9 kg/m^2^)	9.02 ± 2.23	0.68 ± 1.16
	9.00 (8.00–11.0)	0.00 (0.00–1.00)
Obesity (BMI ≥ 30.0 kg/m^2^)	8.80 ± 2.25	1.04 ± 1.15
	9.00 (7.00–10.0)	0.00 (0.00–2.00)
*p*-value	0.727	< 0.001
**Menopause**		
No	9.03 ± 2.36	0.60 ± 1.18
	9.00 (8.00–11.0)	0.00 (0.00–1.00)
Yes	8.78 ± 2.16	0.85 ± 1.29
	9.00 (7.00–10.0)	0.00 (0.00–2.00)
*p*-value	0.152	0.026
**Experienced constipation**		
No	8.92 ± 2.32	0.57 ± 1.13
	9.00 (7.00–11.0)	0.00 (0.00–0.25)
Yes	9.11 ± 2.24	1.19 ± 1.44
	9.00 (8.00–11.0)	0.00 (0.00–2.00)
*p*-value	0.569	< 0.001
**Experienced chronic cough**		
No	8.94 ± 2.32	0.66 ± 1.22
	9.00 (7.00–11.0)	0.00 (0.00–1.00)
Yes	9.14 ± 2.08	1.05 ± 1.21
	9.00 (7.75–11.0)	1.00 (0.00–2.00)
*p*-value	0.741	0.022
**Used diuretics**		
No	8.96 ± 2.31	0.67 ± 1.23
	9.00 (7.00–11.0)	0.00-(0.00–1.00)
Yes	8.84 ± 2.27	0.79 ± 1.08
	9.00 (7.00–11.0)	0.00 (0.00–2.00)
*p*-value	0.842	0.325
**Drink a lot of tea and coffee**		
No	9.04 ± 2.26	0.54 ± 1.08
	9.00 (8.00–11.0)	0.00 (0.00–1.00)
Yes	8.87 ± 2.35	0.81 ± 1.32
	9.00 (7.00–11.0)	0.00 (0.00–1.00)
*p*-value	0.600	0.055
**Performed hip surgery**		
No	8.89 ± 2.34	0.64 ± 1.19
	9.00 (7.00–11.0)	0.00 (0.00–1.00)
Yes	9.34 ± 2.01	0.96 ± 1.39
	10.0 (8.00–11.0)	0.00 (0.00–2.00)
*p*-value	0.182	0.039
**Performed a surgical procedure in the abdominal region**		
No	9.05 ± 2.38	0.66 ± 1.21
	9.00 (8.00–11.0)	0.00 (0.00–1.00)
Yes	8.71 ± 2.11	0.72 ± 1.26
	9.00 (7.00–10.0)	0.00 (0.00–1.00)
*p*-value	0.122	0.508
**Diagnosed with diabetes mellitus**		
No	8.96 ± 2.32	0.63 ± 1.19
	9.00 (7.00–11.0)	0.00 (0.00–1.00)
Yes	8.89 ± 2.16	1.04 ± 1.40
	9.00 (7.00–11.0)	0.00 (0.00–2.00)
*p*-value	0.625	0.026
**Diagnosed with hypertension**		
No	8.97 ± 2.31	0.63 ± 1.21
	9.00 (8.00–11.0)	0.00 (0.00–1.00)
Yes	8.83 ± 2.28	0.98 ± 1.26
	9.00 (7.00–11.0)	0.00 (0.00–2.00)
*p*-value	0.522	0.011
**Diagnosed with hypercholesterolemia**		
No	9.01 ± 2.40	0.54 ± 1.10
	9.00 (7.00–11.0)	0.00 (0.00–0.00)
Yes	8.72 ± 1.89	1.23 ± 1.49
	9.00 (7.00–10.0)	1.00 (0.00–2.00)
*p*-value	0.146	< 0.001
**History of UTI**		
No	8.90 ± 2.34	0.55 ± 1.11
	9.00 (7.00–11.0)	0.00 (0.00–0.00)
Yes	9.13 ± 2.19	1.12 ± 1.44
	9.00 (7.00–11.0)	0.00 (0.00–2.00)
*p*-value	0.535	< 0.001

### 3.5 Factors associated with urinary incontinence: logistic regression analysis

The multivariate logistic regression model (Table 4) identified several independent factors associated with UI. Compared to women aged 25–35, those aged 46–55 and 56–65 had significantly higher odds of UI (OR = 3.22, 95% CI: 1.57–6.60 and OR = 3.41, 95% CI: 1.23–9.44, respectively). Married women had 2.61 times the odds of UI compared to unmarried women (95% CI: 1.19–5.73). Weight status was a strong predictor, with overweight and obese women having 1.96 (95% CI: 1.11–3.47) and 2.97 (95% CI: 1.66–5.32) times the odds, respectively.

**Table 4:** Logistic regression analysis of factors associated with urinary incontinence (*n* = 394).

**Variable**	**Odds ratio (OR)**	**95% Confidence interval (CI)**	***p*-value**
**Age group**			
25–35 years	*Reference category*		
36–45 years	2.07	0.99–4.36	0.055
46–55 years	3.22	1.57–6.60	0.001
56–65 years	3.41	1.23–9.44	0.018
**Marital status**			
Not married	*Reference category*		
Married	2.61	1.19–5.73	0.017
**Weight status**			
Not overweight/obese (BMI < 25.0 kg/m^2^)	*Reference category*		
Overweight (BMI = 25.0–29.9 kg/m^2^)	1.96	1.11–3.47	0.021
Obesity (BMI ≥ 30.0 kg/m^2^)	2.97	1.66–5.32	< 0.001
**Experienced constipation**			
No	*Reference category*		
Yes	2.20	1.30–3.72	0.003
**Experienced chronic cough**			
No	*Reference category*		
Yes	3.67	1.52–8.85	0.004
**Used diuretics**			
No	*Reference category*		
Yes	2.75	1.09–6.95	0.033
**Diagnosed with hypercholesterolemia**			
No	*Reference category*		
Yes	2.41	1.45–3.99	0.001
**History of UTI**			
No	*Reference category*		
Yes	2.34	1.48–3.92	< 0.001
**Knowledge and practices relate to UI**			
Knowledge related to UI	0.96	0.88–1.06	0.436
Practices related to UI	2.94	2.31–3.72	< 0.001

Significant at 95% confidence level. Only significant models were presented in the table.

Clinical factors such as constipation (OR = 2.20, 95% CI: 1.30–3.72), chronic cough (OR = 3.67, 95% CI: 1.52–8.85), use of diuretics (OR = 2.75, 95% CI: 1.09–6.95), hypercholesterolemia (OR = 2.41, 95% CI: 1.45–3.99), and history of UTI (OR = 2.34, 95% CI: 1.48–3.92) were also significant independent predictors. Furthermore, for every one-point increase in the negative practices score, the odds of having UI increased by 194% (OR = 2.94, 95% CI: 2.31–3.72). Knowledge score was not a significant predictor in the model (OR = 0.96, 95% CI: 0.88–1.06, *p* = 0.436).

## 4 Discussion

This study provides a comprehensive assessment of the prevalence, knowledge, practices, and risk factors associated with urinary incontinence among women in Madinah, Saudi Arabia. The key findings reveal a 30% prevalence of UI, a stark disconnect between general awareness and specific knowledge of UI types, and a profile of risk factors where negative health-seeking practices emerged as the strongest modifiable predictor.

The 30% prevalence of UI found in our study is consistent with rates reported in other parts of Saudi Arabia and the broader Middle East (21, 26, 27), confirming UI as a common, though often unspoken, health issue. The predominance of stress UI symptoms in our sample aligns with global epidemiological data, which often identifies it as the most frequent type in community-dwelling women, particularly those of reproductive and perimenopausal age (4, 28, 29). The considerable reporting of mixed UI symptoms underscores the complex and overlapping pathophysiology that many women experience (30).

A critical finding of this study is the stark discrepancy between general awareness and specific, actionable knowledge. While most women knew that UI is treatable and can be managed, their understanding of the specific types was profoundly low, with recognition rates for stress vs. urge UI and mixed UI at a mere 10.9% and 5.3%, respectively. This gap is clinically significant. Understanding the type of UI is the cornerstone of appropriate treatment; for instance, pelvic floor muscle training is first-line for stress UI, while bladder training and anticholinergics are for urge UI (15, 31). Without this knowledge, women cannot self-identify their symptoms to articulate them to a provider, creating a major barrier to receiving effective, targeted care. The primary source of information being social media (41.6%), rather than healthcare professionals (22.1%), highlights a missed opportunity for structured patient education within the healthcare system (32, 33). It also points to the potential value of creating authoritative, easily accessible online resources to fill this knowledge void (41).

The practices scale revealed detrimental health behaviors, with nearly one in five women hesitant to seek medical help. This treatment hesitancy, driven by embarrassment and stigma, is a well-documented barrier globally (13, 34). Our logistic regression model yielded a crucial insight: while the general knowledge score was not a significant predictor of UI, the negative practices score was the strongest modifiable predictor (OR = 2.94). This suggests that the physiological and clinical risk factors are the primary drivers of developing UI. However, once UI is present, an individual's behavior—specifically, whether they avoid activities, neglect the problem due to embarrassment, or hesitate to seek care—becomes powerfully associated with the reported condition. This creates a vicious cycle where the presence of symptoms, potentially exacerbated by inaction, reinforces negative practices, which in turn prevent effective management and worsen the condition's psychosocial impact (35).

Our risk factor profile is largely consistent with global literature. The powerful graded relationship between BMI and UI odds reinforces compelling evidence linking obesity to increased intra-abdominal pressure and pelvic floor burden (10, 36). With over two-thirds of our sample being overweight or obese, this represents a primary public health target for UI prevention. Furthermore, the strong association with marital status, which serves as a proxy for parity and sexual activity in this cultural context, aligns with data from the Gulf region explicitly linking UI to high parity (37). This finding, considered alongside the reviewer's pertinent observation of Saudi Arabia's dramatic fertility rate decline (from ~8 to ~2 births per woman over recent decades), suggests an important area for future research. Our study, which includes women from a wide age range (25–65 years), indirectly reflects this demographic transition. The significantly higher odds of UI among married women likely capture the effect of higher parity in older generations, suggesting that obstetric history may contribute to a higher population-attributable risk in this population. Longitudinal studies tracking specific parity data across generations are needed to directly elucidate this effect.

The universal barrier of stigma appears to be amplified in the Saudi context by specific cultural factors, including profound modesty, which can deter discussions about pelvic health, especially with male providers (38), and potential fatalistic attitudes that may lead to the normalization of symptoms as an inevitable part of aging or childbirth (39). This is reflected in our data, where only 22.1% of women received information from a healthcare professional, underscoring a critical communication gap that public health initiatives must bridge.

### 4.1 Limitations

This study has several limitations. The cross-sectional design precludes any causal inferences. The use of convenience sampling via social media likely under-represents women with lower education, lower digital literacy, or less access to social media, potentially leading to selection bias. This may result in an underestimation of the true knowledge gaps and an overestimation of general knowledge in the broader population of Madinah. Furthermore, while the high prevalence of overweight and obesity in our sample (68.5%) is consistent with recent national estimates for Saudi women (13), the online recruitment method might have skewed the sample, and thus, the generalizability of our findings to all women in Madinah should be interpreted with caution. Data on UI and comorbidities were self-reported, which is subject to recall and social desirability bias. The questionnaire, though developed through comprehensive literature review and expert validation, was not a previously standardized instrument. An additional limitation is the lack of assessment of UI severity using a validated scale. Information on the frequency and volume of leakage would have provided a more nuanced understanding of the condition's burden. Finally, the assessment of UI and its types was based on self-reported symptoms rather than a clinical diagnosis or objective measures like a pad test. As noted by the reviewer, there can be a discrepancy between volunteered incontinence on a questionnaire and objectively demonstrated leakage, which may lead to prevalence misestimation and UI type misclassification.

## 5 Conclusions

In conclusion, this study establishes that urinary incontinence is a highly prevalent condition among women in Madinah, Saudi Arabia, and is associated with a distinct profile of demographic, clinical, and—most critically—behavioral factors. The identified risk factors, including older age, obesity, and specific comorbidities, provide a clear checklist for healthcare providers to screen high-risk individuals. However, the most significant finding is the critical gap in specific UI knowledge and the strong association between negative health-seeking practices and UI.

To address this, a multi-pronged approach is essential:

1. *Public health campaigns:* develop culturally tailored, social media-savvy campaigns that move beyond general awareness to educate women on the different types of UI and their specific, effective treatments, thereby countering fatalism and empowering informed action.
2. *Clinical practice integration:* healthcare systems should implement routine, sensitive screening for UI in primary care and gynecological settings, particularly for high-risk women (older, obese, multiparous), to proactively break the silence and normalize the conversation.
3. *Specialist service development:* invest in training more female urogynecologists and pelvic health physiotherapists to ensure that when women overcome barriers to seek help, they have access to culturally appropriate and specialized care.

Future studies should also consider exploring the influence of sociocultural factors such as consanguinity on the prevalence and perception of UI, which was beyond the scope of the current investigation.

## Data Availability

The raw data supporting the conclusions of this article will be made available by the authors, without undue reservation.
